# Supplementary material for: Optimizing Pediatric Intermediate Care: Clinical Predictors of Deterioration and Length of Stay in a Tertiary Setting
Source: J Clin Med. 2025 Sep 10;14(18):6398. doi: 10.3390/jcm14186398 (PMC12471059; doi:10.3390/jcm14186398)
Supplement: Supplementary file 1 [file jcm-14-06398-s001.zip › jcm-3823301-supplementary.pdf]

1. INDICE

1. INDICE 1
2. STORIA DEL DOCUMENTO 3
3. SCOPO E CAMPO D'APPLICAZIONE 3
   3.1 Finalità del documento 3
   3.2 Principi generali 4
   3.3 Organico e responsabilità 4
4. DEFINIZIONI, ACRONIMI E ABBREVIAZIONI 5
5. MODALITA' OPERATIVE E RESPONSABILITA' 6
   5.1 Criteri di Ingresso in Terapia Intensiva (UOC Terapia Intensiva Neonatale e Pediatrica) 6
   5.2 Procedura di trasferimento del paziente in Terapia Intensiva 6
   5.3 Patologie e apparati di pertinenza della Terapia Intensiva 6
      5.3.1 Apparato Respiratorio 6
      5.3.2 Apparato Cardiovascolare 7
      5.3.3 Sistema Nervoso 7
      5.3.4 Ematologia/oncologia 8
      5.3.5 Sistema Endocrino/Metabolico 8
      5.3.6 Apparato Gastrointestinale 9
      5.3.7 Apparato Urinario 9
      5.3.8 Paziente Chirurgico 9
      5.3.9 Paziente con compromissione multisistemica o altro 10
   5.4 Necessità di speciali supporti tecnologici intensivi 10
      5.4.1 Necessita` di supporti extracorporei 10
   5.5 Criteri di dimissibilita` dalla Terapia Intensiva (UOC Terapia Intensiva Neonatale e Pediatrica) 10
      5.5.1 Criteri clinici 10
      5.5.2 Modalità operative del trasferimento 11
   5.6 Criteri di Ingresso in Terapia Semintensiva 12
      5.6.1 Apparato Respiratorio 12

Questo documento è di proprietà dell'IRCCS "GIANNINA GASLINI", ogni riproduzione non autorizzata dallo stesso è vietata

5.6.2 Apparato Cardiovascolare 12
5.6.3 Sistema Nervoso 13
5.6.4 Ematologia/Oncologia 13
5.6.5 Sistema Endocrino/Metabolico 13
5.6.6 Apparato Gastointestinale 14
5.6.7 Apparato Urinario 14
5.6.8 Paziente Chirurgico 14
5.6.9 Paziente con compromissione multisistemica o altro 15
5.7 Criteri di dimissibilita` dalla Terapia Semintensiva 15
5.7.1 Criteri clinici 15
5.7.2 Modalità operative del trasferimento 16
6. DISTRIBUZIONE E ARCHIVIAZIONE 16
7. RIFERIMENTI 16
8. ALLEGATI 16

Questo documento è di proprietà dell'IRCCS "GIANNINA GASLINI", ogni riproduzione non autorizzata dallo stesso è vietata

2. STORIA DEL DOCUMENTO

Rev.	Data	Redazione	Revisione	Verifica	Approvazione	Modifiche
0	13/07/2007	U.O.C. Anestesia e Rianimazione	RAQ	RSGQ	Direzione Sanitaria<br>U.O. Controllo di Gestione e Servizio Qualità	Prima emissione
1	26/04/2010	U.O.C. Anestesia e Rianimazione	RAQ	RSGQ	Direzione Sanitaria<br>U.O. Controllo di Gestione e Servizio Qualità	Revisione Periodica
2	21/05/2013	U.O.C. Anestesia e Rianimazione	RAQ	RSGQ	Direzione Sanitaria<br>U.O. Controllo di Gestione e Servizio Qualità	Revisione Periodica
3	05/08/2016	U.O.C. Anestesia e Rianimazione	RAQ	RSGQ	Direzione Sanitaria<br>U.O. Controllo di Gestione e Servizio Qualità	Revisione Periodica
4	02/10/2019	U.O.C. Terapia Intensiva Neonatale e Pediatrica	RAQ	RSGQ	Direzione Sanitaria<br>U.O. Controllo di Gestione e Servizio Qualità	Modifiche par. 3.1, 3.2, 3.3, 5.1, 5.1.1, 5.2, 5.2.2, 5.3.
5	02/02/2021	U.O.C. Terapia Intensiva Neonatale e Pediatrica	RAQ	RSGQ	Direzione Sanitaria<br>U.O. Controllo di Gestione e Servizio Qualità	Revisione Periodica
6	15/01/2024	Elisabetta Lampugnani<br>UOC Terapia Intensiva Neonatale e Pediatrica	Sara Palmerino<br>Ines Lorenzi<br>UOC Governo Clinico – Servizio Qualità<br>Andrea Moscatelli<br>Direttore Dipartimento DEA	Giuseppe Spiga<br>RSGQ	Raffaele Spiazzi<br>Direttore Sanitario<br>**FIRMATO DIGITALMENTE: Direttore Sanitario - Raffaele Spiazzi (03/05/2024 13:45)**<br>[firma_digitale]	Revisione periodica

3. SCOPO E CAMPO D'APPLICAZIONE

3.1 Finalità del documento

Questa procedura si applica alla Terapia Intensiva e Semintensiva pediatrica.

Questo documento è di proprietà dell'IRCCS "GIANNINA GASLINI", ogni riproduzione non autorizzata dallo stesso è vietata

In questo documento vengono illustrati i criteri di ricovero in Terapia Intensiva (UOC Terapia Intensiva Neonatale e Pediatrica) e Semintensiva (Terapia Semintensiva Primo Piano DEA, degenza Semintensiva della UOC Cardiologia-Cardiochirurgia e degenza Semintensiva dell'UOSD Centro di Trapianto di Midollo Osseo e Cellule Staminali). Sono definite le dotazioni organiche raccomandate e gli ambiti di responsabilità. Vengono, inoltre, indicate le modalità di interazione tra UOC Terapia Intensiva Neonatale e Pediatrica e UOC Patologia Neonatale - Terapia Intensiva Neonatale per quanto riguarda il neonato chirurgico, il neonato a rischio di supporti extra-corporei (ECMO, Continuous Renal Replacement Therapy - CRRT), il neonato cardiopatico (cardiologico-cardiochirurgico) o con grave compromissione emodinamica e quello affetto da patologia malformativa/ostruttiva delle vie aeree (Team delle Vie Aeree).

3.2 Principi generali

- Sono ricoverati in Terapia Intensiva, UOC Terapia Intensiva Neonatale e Pediatrica, pazienti instabili con deficit di una o più funzioni d'organo.

- Pazienti stabili ma con rischio potenziale di sviluppare un deterioramento clinico significativo, che richiedano monitoraggi frequenti dei parametri vitali, non di tipo intensivo, ed elevata sorveglianza/frequenti interventi da parte del personale infermieristico sono ricoverati presso i vari settori semintensivi dell'Istituto. Il settore Semintensivo di pertinenza viene identificato a seconda della patologia di base e delle competenze specifiche delle singole aree di degenza (settore semintensivo UOC Terapia Intensiva Neonatale e Pediatrica, Terapia Semintensiva Primo Piano DEA, degenza Semintensiva della UOC Cardiologia-Cardiochirurgia e degenza Semintensiva dell'UOSD Centro di Trapianto di Midollo Osseo e Cellule Staminali).

- Sono ricoverati in Terapia Intensiva, presso l'UOC Terapia Intensiva Neonatale e Pediatrica, neonati chirurgici, neonati a rischio di supporti extracorporei (ECMO, CRRT), neonati cardiopatici (cardiologici-cardiochirurgici) o con grave compromissione emodinamica, tale da richiedere approccio specialistico (Terapia Intensiva Cardiologica), neonati affetti da patologia malformativa/ostruttiva delle vie aeree (Team delle Vie Aeree). Superata la problematica specifica, che ne indica il ricovero presso l'UOC Terapia Intensiva Neonatale e Pediatrica, il paziente "neonato" ha come luogo elettivo di ricovero l'UOC Patologia Neonatale-Terapia Intensiva Neonatale, a meno che non siano richieste competenze specifiche, nel qual caso il ricovero elettivo avverrà presso la UOC Terapia Intensiva Neonatale e Pediatrica.

3.3 Organico e responsabilità

In Terapia Intensiva (UOC Terapia Intensiva Neonatale e Pediatrica) è presente h24 una guardia attiva composta da due medici. I medici di guardia sono responsabili dei pazienti ricoverati (case manager) e coordinano l'attività degli specialisti, con i quali condividono i programmi diagnostico-terapeutici. La

Questo documento è di proprietà dell'IRCCS "GIANNINA GASLINI", ogni riproduzione non autorizzata dallo stesso è vietata

terapia Intensiva è attualmente dotata di 16 posti letto con un rapporto infermiere/paziente variabile tra 1:3, 1:2, 1:1 ed eccezionalmente 2:1 (paziente in ECMO con CRRT). Idealmente viene raccomandato un rapporto 1:2. I posti letto di Terapia Intensiva sono espandibili a 20.

Presso i settori Semintensivi non è generalmente prevista guardia attiva h24 (presenza del medico specialista h12, pronta disponibilità h24); il case manager (medico tutor) è lo specialista di riferimento che coordina l'attività di tutti gli altri specialisti. La continuità assistenziale è comunque garantita h24 dal medico di guardia interna. La Terapia Semintensiva situata al Primo Piano del DEA (12 posti letto) è dotata, all'equilibrio, di un organico medico di 6-7 unità. È garantita la presenza di un medico pediatra in turni di guardia h24, 7 giorni/7. Limitatamente a quest'area di degenza, è previsto un programma di affiancamento con medico pediatra in formazione a copertura delle 24 ore, 7 giorni/7. Nelle ore notturne e nei festivi, in casi particolari e per problematiche urgenti, i medici di guardia presso l'UOC Terapia Intensiva Neonatale e Pediatrica sono a disposizione per fornire supporto agli altri settori Semintensivi, in attesa dell'intervento dei medici reperibili di riferimento. In caso di urgenza di non diretta competenza intensivistica, qualora i medici della Terapia Intensiva fossero contemporaneamente occupati, potrà essere chiamato in supporto il medico pediatra di guardia interna. Limitatamente alla Terapia Semintensiva, Primo Piano DEA, il case manager del paziente (medico tutor) è il medico pediatra in servizio nel settore. Per problematiche specifiche si farà riferimento agli specialisti della condizione clinica prevalente, che dovranno comunque ritenersi sempre a disposizione, nell'ambito dei propri turni di servizio e reperibilità.

È raccomandato un rapporto infermiere/paziente di 1:3-1:4.

4. DEFINIZIONI, ACRONIMI E ABBREVIAZIONI

UOC: Unità Operativa Complessa

UOSD: Unità Operativa Semplice Dipartimentale

DEA: Dipartimento Emergenza e Accettazione

ECMO: Extra Corporeal Membrane Oxygenation

CRRT: Continuous Renal Replacement Therapy

IMV: Ventilazione Meccanica Invasiva

NIV: Ventilazione Non Invasiva

GB: Globuli Bianchi

LFVVECO_2R: Low Flow Veno-Venous Extracorporeal CO2 Removal

Questo documento è di proprietà dell'IRCCS "GIANNINA GASLINI", ogni riproduzione non autorizzata dallo stesso è vietata

CPFA: Coupled Plasmafiltration – Adsorption

CEC: Circolazione Extra Corporea

FiO_2: Frazione inspirata di Ossigeno

5. MODALITA' OPERATIVE E RESPONSABILITA'

5.1 Criteri di Ingresso in Terapia Intensiva (UOC Terapia Intensiva Neonatale e Pediatrica)

- Sono ricoverati in Terapia Intensiva pazienti **instabili** con deficit di una o più funzioni d'organo.

- Il paziente acuto, nel quale è prevedibile una rapida progressione della gravità dei deficit d'organo viene ricoverato in Terapia Intensiva.

- Sono ricoverati in terapia intensiva pazienti post-chirurgici che richiedano monitoraggi di tipo intensivo.

5.2 Procedura di trasferimento del paziente in Terapia Intensiva

Il trasferimento del paziente dai reparti di degenza all'UOC Terapia Intensiva Neonatale e Pediatrica avviene sotto la responsabilità del medico anestesista rianimatore dell'UOC Terapia Intensiva Neonatale e Pediatrica, coadiuvato da un'infermiera della UO di provenienza del paziente. Il trasferimento deve prevedere sempre un passaggio di consegne scritte a cura del medico del reparto di provenienza (ovvero a cura del medico di guardia interna, qualora non presente il medico di reparto), comprendenti una sintetica epicrisi del caso, eventuali esami eseguiti in urgenza, terapie effettuate e terapie in atto ed ogni altra informazione utile all'inquadramento ed alla migliore prosecuzione delle cure. In caso di discussione collegiale sull'opportunità del trasferimento presso l'UOC Terapia Intensiva Neonatale e Pediatrica ed in presenza di pareri discordanti la decisione finale è di competenza del medico anestesista rianimatore.

5.3 Patologie e apparati di pertinenza della Terapia Intensiva

5.3.1 Apparato Respiratorio

Pazienti con patologia polmonare o delle vie aeree, grave o potenzialmente pericolosa per la vita. Le condizioni prevalenti ma non esclusive comprendono:

- Intubazione endotracheale o potenziale necessità di intubazione endotracheale d'urgenza e ventilazione meccanica invasiva (IMV), indipendentemente dall'eziologia.

Questo documento è di proprietà dell'IRCCS "GIANNINA GASLINI", ogni riproduzione non autorizzata dallo stesso è vietata

- Ventilazione non invasiva (NIV) avviata acutamente con FiO_2 maggiore 0,4.
- Patologia grave, rapidamente progressiva dei polmoni, delle vie aeree alte o basse, con rischio di evoluzione verso l'insufficienza respiratoria e/o totale ostruzione delle vie aeree.
- Elevato fabbisogno di ossigeno (FiO_2 >0.5), indipendentemente dall'eziologia.
- Recente confezionamento di tracheotomia, con necessità di ventilazione meccanica.
- Barotrauma acuto con compromissione delle alte o basse vie aeree (pneumotorace, pneumomediastino, etc).

5.3.2 Apparato Cardiovascolare

Pazienti con patologia cardiovascolare grave, minacciosa per la vita, o instabile. Le condizioni prevalenti ma non esclusive comprendono:

- Shock o sepsi grave.
- Status post-rianimazione cardiopolmonare.
- Aritmie minacciose per la vita.
- Scompenso cardiaco congestizio instabile, con o senza necessità di IMV/NIV e non.
- Patologia cardiaca congenita caratterizzata da instabilità cardio-respiratoria.
- Necessità di monitoraggio emodinamico avanzato (include ma non e` limitato a pressione arteriosa polmonare o arteriosa cruenta e venosa centrale).
- Necessità di stimolazione cardiaca temporanea.
- Post-operatorio di procedure cardiovascolari o toraciche ad alto rischio.

5.3.3 Sistema Nervoso

Pazienti con patologia neurologica instabile, o in pericolo di vita imminente o potenziale. Le condizioni prevalenti ma non esclusive comprendono:

- Crisi epilettiche, non rispondenti alla terapia o che richiedono infusione continua di agenti anticonvulsivanti (stato di male epilettico).
- Stroke, malformazioni vascolari cerebrali (es.: VGAM).
- Alterazioni del sensorio acute e gravi in cui il deterioramento o la depressione neurologica siano probabili o imprevedibili, oppure coma con possibile compromissione delle vie aeree.
- Post-operatorio di procedure neurochirurgiche che richiedano monitoraggio invasivo o stretta osservazione.
- Infiammazioni o infezioni acute di midollo spinale, meningi o encefalo con depressione neurologica, alterazioni metaboliche ed ormonali, compromissione respiratoria o emodinamica o possibile aumento della pressione intracranica.
- Trauma cranico a rischio di incremento della pressione intracranica.

Questo documento è di proprietà dell'IRCCS "GIANNINA GASLINI", ogni riproduzione non autorizzata dallo stesso è vietata

- Disfunzione neuromuscolare acuta progressiva con o senza alterazione del sensorio, che richieda monitoraggio cardiovascolare e/o supporto respiratorio (invasivo e non).
- Compressione spinale.
- Iniziale decorso postoperatorio successivo a posizionamento di dispositivo di drenaggio ventricolare esterno in pazienti a rischio (prematuri, alterazione dello stato di coscienza).
- Necessità di monitoraggio della pressione intracranica.
- Encefalopatia ipossico-ischemica oltre l'età neonatale, con possibile indicazione a ipotermia terapeutica.

5.3.4 Ematologia/oncologia

Pazienti con patologie ematologiche o oncologiche instabili o minacciose per la vita o sanguinamenti attivi con pericolo di vita. Le condizioni prevalenti ma non esclusive comprendono:

- Reazioni trasfusionali.
- Plasmaferesi o leucoferesi in pazienti clinicamente instabili.
- Coagulopatia acuta grave.
- Anemia grave con compromissione emodinamica e/o respiratoria.
- Tumori o masse che comprimono o minacciano di comprimere vasi o organi vitali o le vie aeree.
- Rischio di grave sindrome da lisi tumorale a seguito di inizio di chemioterapia.
- Sindrome da iperviscosita` con GB > 200.000-300.000, ma anche con livelli ematici inferiori in caso di segni neurologici o gravi alterazioni metaboliche (iperuricemia, iperpotassiemia).

5.3.5 Sistema Endocrino/Metabolico

Pazienti con patologia metabolica o endocrina grave, pericolosa per la vita o instabile. Le condizioni prevalenti ma non esclusive comprendono:

- Chetoacidosi diabetica grave in presenza di compromissione emodinamica, respiratoria o neurologica.
- Gravi alterazioni idro-elettrolitiche:
  - Iperpotassiemia, che richieda monitoraggio cardiaco ed interventi terapeutici in urgenza.
  - grave ipo o ipernatriemia.
  - grave ipo o ipercalcemia.
- Grave ipoglicemia che richieda monitoraggio intensivo.
- Grave acidosi metabolica che richieda infusione di alcalinizzanti.
- Alterazioni idro-elettrolitiche o dell'equilibrio acido-base che richiedano monitoraggio intensivo ed interventi terapeutici complessi (es.: CRRT).
- Gravi alterazioni del bilancio idrico che richiedano interventi terapeutici complessi (es.: CRRT).

Questo documento è di proprietà dell'IRCCS "GIANNINA GASLINI", ogni riproduzione non autorizzata dallo stesso è vietata

- Errori congeniti del metabolismo con deterioramento acuto che richieda supporto respiratorio, dialisi acuta, emoperfusione, gestione dell'ipertensione endocranica, o supporto inotropo.

5.3.6 Apparato Gastrointestinale

Pazienti con patologia gastrointestinale pericolosa per la vita o instabile. Le condizioni prevalenti ma non esclusive comprendono:

- Emorragia gastrointestinale acuta grave con instabilità emodinamica o respiratoria.
- Post-operatorio di endoscopia urgente per rimozione di corpi estranei nel caso in cui non possa essere garantita o debba essere monitorizzata la stabilità delle vie aeree, qualora il rischio di ostruzione sia elevato o in caso di ingestione di button battery.
- Ingestione di caustici.
- Insufficienza epatica acuta che possa portare a coma, alterazioni emocoagulative, instabilità emodinamica o respiratoria.

5.3.7 Apparato Urinario

Pazienti con patologia renale pericolosa per la vita o instabile. Le condizioni prevalenti ma non esclusive comprendono:

- Insufficienza renale acuta/cronica con compromissione cardio-respiratoria e metabolica grave.
- Necessità di emodialisi, dialisi peritoneale, o di altre tecniche di sostituzione renale continua (CRRT) nel paziente instabile.
- Rabdomiolisi acuta con insufficienza renale.

5.3.8 Paziente Chirurgico

Pazienti in fase post-operatoria (qualsiasi chirurgia) che richiedono monitoraggio frequente e possibili trattamenti intensivi. Le condizioni prevalenti ma non esclusive comprendono:

- Chirurgia cardiovascolare
- Procedure di cardiologia e radiologia intervenzionale
- Chirurgia toracica
- Neurochirurgia
- Chirurgia otorinolaringoiatrica o delle vie aree
- Chirurgia cranio-faciale
- Chirurgia ortopedica e della colonna
- Chirurgia generale maggiore
- Trapianti d'organo
- Politrauma
- Perdite ematiche maggiori in corso di chirurgia o nell'immediato post-operatorio

Questo documento è di proprietà dell'IRCCS "GIANNINA GASLINI", ogni riproduzione non autorizzata dallo stesso è vietata

5.3.9 Paziente con compromissione multisistemica o altro

Pazienti con patologia multisistemica pericolosa per la vita o instabile. Le condizioni prevalenti ma non esclusive comprendono:

- Intossicazioni e overdose di farmaci con potenziale scompenso acuto dei maggiori sistemi d'organo.
- Sindrome da disfunzione multiorgano.
- Ipertermia maligna sospetta o documentata.
- Danni da elettrocuzione o da altre cause domestiche o ambientali (es. da fulmine).
- Ustioni di estensione > 10% della superficie corporea con instabilità emodinamica e/o respiratoria.
- Patologie infettive altamente diffusive (es.: meningococcemia, tubercolosi, COVID-19).

Presso la UOC Terapia Intensiva Neonatale e Pediatrica vengono centralizzati tutti i pazienti intensivi COVID-19 positivi o a rischio COVID-19, indipendentemente dall'età pediatrica, compreso il neonato prematuro.

5.4 Necessità di speciali supporti tecnologici intensivi

Condizioni che necessitano dell'applicazione di speciali supporti tecnologici, monitoraggi, interventi complessi, o trattamenti farmacologici, che superano i limiti degli indirizzi di cura per il singolo paziente in reparti non intensivi o semi-intensivi.

5.4.1 Necessita` di supporti extracorporei

- ECMO (extracorporeal membrane oxygenation).
- LFVVECO_2R (low flow veno-venous extracorporeal CO_2 removal).
- CRRT (continuous renal replacement therapy).
- Plasmaferesi nella Multiorgan Dysfunction Syndrome.
- CPFA (coupled plasmafiltration-adsorption).

5.5 Criteri di dimissibilita` dalla Terapia Intensiva (UOC Terapia Intensiva Neonatale e Pediatrica)

5.5.1 Criteri clinici

Il trasferimento viene programmato non appena le condizioni cliniche del paziente lo consentano, per risoluzione delle insufficienze d'organo e per il venir meno delle necessità tecnologiche e di monitoraggio che hanno richiesto il ricovero in Terapia Intensiva. La dimissibilità del paziente è definita sotto la responsabilità ed in base al giudizio del medico Intensivista, tenendo conto dei seguenti criteri:

- Stabilità emodinamica.

Questo documento è di proprietà dell'IRCCS "GIANNINA GASLINI", ogni riproduzione non autorizzata dallo stesso è vietata

- Stabilità respiratoria (paziente estubato, con adeguata pervietà delle vie aeree, eventualmente con supporto ventilatorio non invasivo stabile).
- Fabbisogno di ossigeno stabile con nasocannule o nasocannule ad alto flusso. Generalmente con FiO_2 non superiore a 0.45 (escluse condizioni croniche stabilizzate).
- Non necessità di monitoraggio emodinamico avanzato (generalmente non vengono dimessi pazienti con monitoraggio delle pressioni cruente, ad esclusione del settore Semi-Intensivo della Cardiologia).
- Minimo fabbisogno di farmaci vasoattivi (escluse condizioni croniche e stabilizzate).
- Aritmie controllate o che non rappresentino un pericolo di vita per il paziente.
- Non necessità del monitoraggio continuo della pressione intracranica.
- Stabilità neurologica, convulsioni controllate dalla terapia (escluse condizioni croniche di epilessia farmaco-resistente).
- Paziente con supporto ventilatorio non invasivo, stabile, superata la fase acuta di instabilità.
- Paziente in emodialisi o dialisi peritoneale, superata la fase acuta di instabilità.
- Pazienti con tracheotomia stabilizzata.

Pazienti con patologia a prognosi infausta quando, a seguito di una valutazione collegiale con coinvolgimento della famiglia e dello psicologo di reparto, si ritenga futile/accanimento terapeutico la prosecuzione delle cure in ambiente intensivo.

5.5.2 Modalità operative del trasferimento

In caso di trasferimento dalla Terapia Intensiva la procedura prevede:

a) Il reparto di destinazione viene identificato in base a:

- patologia di base;
- tipologia del monitoraggio clinico ed infermieristico necessario;
- prossimità alla Terapia Intensiva (se necessario).

b) Vengono presi contatti tra il medico della UOC Terapia Intensiva Neonatale e Pediatrica ed il medico della U.O. di destinazione e, ove pertinente, si procede ad una valutazione congiunta del paziente stesso. In caso di temporanea indisponibilità di posto letto presso il reparto di destinazione, sarà cura del medico del reparto stesso attivarsi per adeguare la recettività del reparto nel più breve tempo possibile. Preliminarmente al trasferimento il medico della Terapia Intensiva predispone una sintetica epicrisi del caso, dove sia data evidenza dei problemi aperti e del programma di cura (terapie in atto). Il trasporto durante il trasferimento avviene a cura delle infermiere del reparto ricevente, eventualmente coadiuvate dal medico di reparto per i trasferimenti ai settori Semi-Intensivi. In caso di trasferimento di pazienti fra Terapia Intensiva (UOC Terapia Intensiva Neonatale e Pediatrica) e UOC Patologia Neonatale – Terapia Intensiva Neonatale, il trasporto è garantito da personale dell'UOC Terapia Intensiva Neonatale e

Questo documento è di proprietà dell'IRCCS "GIANNINA GASLINI", ogni riproduzione non autorizzata dallo stesso è vietata

Pediatrica, viceversa in caso di trasferimento da UOC Patologia Neonatale-Terapia Intensiva Neonatale a UOC Terapia Intensiva Neonatale e Pediatrica.
Deve essere predisposto un sistema di attento monitoraggio dei tempi di dimissione dalle Terapie Intensive, favorendo i flussi in uscita, nell'ottica di ottimizzare l'utilizzo delle risorse intensive disponibili in Istituto. Eventuali ritardi andranno segnalati tempestivamente alla Direzione Sanitaria e al Bed Manager.

5.6 Criteri di Ingresso in Terapia Semintensiva

- Sono ricoverati in Terapia Semintensiva pazienti **stabili** ma con **rischio potenziale di sviluppare un deterioramento clinico significativo**, che richiedano monitoraggi frequenti dei parametri vitali, non di tipo intensivo, ed elevata sorveglianza/frequenti interventi da parte del personale infermieristico.
- Il settore Semitensivo di pertinenza viene identificato a seconda della patologia di base e delle competenze specifiche delle singole aree di degenza (Terapia Semintensiva Primo Piano DEA, degenza Semintensiva della UOC Cardiologia-Cardiochirurgia e degenza Semintensiva dell'UOSD Centro di Trapianto di Midollo Osseo e Cellule Staminali).

5.6.1 Apparato Respiratorio

Pazienti con patologia polmonare o delle vie aeree di grado moderato che richiedano un approccio multidisciplinare e monitoraggi frequenti. Le condizioni prevalenti ma non esclusive comprendono:

- Pazienti a rischio potenziale di intubazione tracheale.
- Pazienti con patologia polmonare evolutiva, con interessamento delle alte o basse vie aeree, di grado moderato a rischi di sviluppare sintomatologia ostruttiva e/o insufficienza respiratoria grave.
- Pazienti che richiedano una $FiO_2 \geq$ 0,35-0,45, indipendentemente dalla causa.
- Pazienti che richiedano un supporto ventilatorio non invasivo o tramite cannula tracheostomica in condizioni di stabilita`.
- Pazienti con una tracheotomia stabile.
- Pazienti che richiedano terapia aerosolica ad intervalli inferiori alle 2 ore.
- Pazienti a rischio ALTE o che richiedano studi polisonnografici (OSAS) o monitoraggio cardio-respiratorio.

5.6.2 Apparato Cardiovascolare

Pazienti con patologia cardiovascolare di grado moderato che richiedano un approccio multidisciplinare e monitoraggi frequenti. Le condizioni prevalenti ma non esclusive comprendono:

- Pazienti con aritmie non ad imminente pericolo per la vita che possano richiedere o meno cardioversione.
- Pazienti con cardiopatie non ad imminente pericolo per la vita che richiedano un supporto stabilizzato con farmaci vasoattivi.

Questo documento è di proprietà dell'IRCCS "GIANNINA GASLINI", ogni riproduzione non autorizzata dallo stesso è vietata

- Pazienti stabili che sono stati sottoposti a procedure cardiochirurgiche (ad esclusione dell'immediato post-CEC) o di cardiologia intervenzionale.

5.6.3 Sistema Nervoso

Pazienti con patologia neurologica non ad imminente pericolo per la vita, che richieda un approccio multidisciplinare e monitoraggi frequenti. Le condizioni prevalenti ma non esclusive comprendono:

- Pazienti con convulsioni, controllate dalla terapia, emodinamicamente stabili ma a rischio di compromissione respiratoria, che richiedano monitoraggio cardiorespiratorio continuo.
- Pazienti con alterazioni del sensorio nei quali è improbabile la progressione della compromissione neurologica, senza depressione della funzione respiratoria/riflessi di protezione delle vie aeree o compromissione emodinamica.
- Pazienti con infezione/infiammazione del sistema nervoso centrale con alterazioni del sensorio nei quali è improbabile la progressione della compromissione neurologica, senza depressione della funzione respiratoria/riflessi di protezione delle vie aeree o compromissione emodinamica.
- Pazienti con trauma cranico senza segni o sintomi neurologici evolutivi.
- Pazienti con compromissione neuromuscolare, in assenza di alterazioni emodinamiche che possano richiedere un supporto ventilatorio non invasivo stabile, senza alterazioni del sensorio.

5.6.4 Ematologia/Oncologia

Pazienti affetti da patologie emato-oncologiche potenzialmente instabili o sanguinamenti non pericolosi per la vita che richiedano un approccio multidisciplinare e monitoraggi frequenti. Le condizioni prevalenti ma non esclusive comprendono:

- Pazienti affetti da anemia grave, in assenza di compromissione cardio-respiratoria.
- Pazienti affetti da trombocitopenia, anemia, neutropenia o tumori solidi a rischio di compromissione cardio-respiratoria con necessità di stretto monitoraggio dei parametri vitali.
- Altre condizioni tipiche del paziente emato-oncologico (sindrome da lisi tumorale, terapie con farmaci biologici, GVH grave, stato settico), a rischio di insufficienza d'organo con necessità di monitoraggio dei parametri vitali o di terapie complesse.

5.6.5 Sistema Endocrino/Metabolico

Pazienti affetti da patologie endocrino-metaboliche potenzialmente instabili che richiedano un approccio multidisciplinare e monitoraggi frequenti. Le condizioni prevalenti ma non esclusive comprendono:

- Pazienti affetti da chetoacidosi diabetica moderata (glicemia $<$ 500 mg/dL, pH $\geq$ 7.20), con necessità di infusione continua di insulina, in assenza di alterazioni del sensorio.
- Pazienti con alterazioni elettrolitiche e/o metaboliche di grado moderato, tali da richiedere monitoraggio cardiologico e interventi terapeutici:

Questo documento è di proprietà dell'IRCCS "GIANNINA GASLINI", ogni riproduzione non autorizzata dallo stesso è vietata

- Ipopotassiemia (K^+ < 2.0 mEq/L), iperpotassiemia (K^+ > 6.0 mEq/L).
- Iper- ed iponatriemia con alterazione dello stato di coscienza e/o convulsioni.
- Ipo-ipercalcemia.
- Ipo-iperglicemia.
- Acidosi metabolica di grado moderato con necessità di infusione di alcalinizzanti.
- Errori congeniti del metabolismo che richiedano monitoraggio cardio-respiratorio.

5.6.6 Apparato Gastointestinale

Pazienti affetti da patologie gastrointestinali potenzialmente instabili che richiedano un approccio multidisciplinare e monitoraggi frequenti. Le condizioni prevalenti ma non esclusive comprendono:

- Pazienti affetti da sanguinamento gastrointestinale, in assenza di instabilità cardiorespiratoria.
- Pazienti che hanno ingerito corpi estranei o che richiedano endoscopia urgente, in assenza di instabilità cardiorespiratoria.
- Pazienti con insufficienza gastrointestinale o epatobiliare, in assenza di alterazione dello stato di coscienza o di instabilità cardiorespiratoria.

5.6.7 Apparato Urinario

Pazienti affetti da patologie dell'apparato urinario potenzialmente instabili che richiedano un approccio multidisciplinare e monitoraggi frequenti. Le condizioni prevalenti ma non esclusive comprendono:

- Pazienti affetti da ipertensione arteriosa, in assenza di convulsioni o encefalopatia o altri sintomi di scompenso cardiorespiratorio, che richiedano monitoraggi e terapie frequenti (per via orale e/o endovenosa).
- Pazienti con sindrome nefrosica non complicata, caratterizzata da ipertensione e che richieda monitoraggi e/o terapie frequenti.
- Pazienti affetti da insufficienza renale, in assenza di instabilita` cardiorespiratoria.
- Pazienti che richiedano dialisi peritoneale o emodialisi cronica.

5.6.8 Paziente Chirurgico

Pazienti affetti sottoposti ad intervento chirurgico che richiedano un approccio multidisciplinare e monitoraggi frequenti in assenza di compromissione cardiorespiratoria o dello stato di coscienza. Le condizioni prevalenti ma non esclusive comprendono:

- cardiochirurgia;
- chirurgia toracica;
- neurochirurgia;
- chirurgia delle vie aeree;
- chirurgia craniofacciale;
- chirurgia addominale;
- chirurgia ortopedica;

Questo documento è di proprietà dell'IRCCS "GIANNINA GASLINI", ogni riproduzione non autorizzata dallo stesso è vietata

- politrauma.

5.6.9 Paziente con compromissione multisistemica o altro

Pazienti con patologia multisistemica potenzialmente instabile che richiedano un approccio multidisciplinare e monitoraggi frequenti. Le condizioni prevalenti ma non esclusive comprendono:

- Pazienti dipendenti da tecnologie/presidi complessi:
  - assistenza respiratoria non invasiva in condizioni di stabilita`;
  - tracheostomia stabilizzata;
  - drenaggi pleurici e/o pericardici in assenza di instabilita` cardiorespiratoria;
  - terapie mediche complesse.
- Pazienti vittima di avvelenamento da sostanze tossiche, in assenza di compromissione cardiorespiratoria, che richiedano stretto monitoraggio.
- Patologie infettive altamente diffusive (es.: meningococcemia, tubercolosi, COVID-19).

Presso la Terapia Semintensiva Primo Piano DEA vengono centralizzati tutti i pazienti semi-intensivi COVID-19 positivi o a rischio COVID-19, indipendentemente dall'età pediatrica, compreso il neonato prematuro.

5.7 Criteri di dimissibilita` dalla Terapia Semintensiva

5.7.1 Criteri clinici

La dimissione in degenza ordinaria verra` presa in considerazione ad avvenuta risoluzione delle condizioni fisiopatologiche che hanno indicato il ricovero nel settore Semi-Intensivo, qualora non siano piu` necessari monitoraggi ed interventi terapeutici frequenti. Il paziente e` dimissibile dalla Terapia Semintensiva se sono soddisfatti i seguenti criteri:

- Parametri emodinamici stabili per almeno 6-12 ore.
- Condizione respiratoria stabile evidenziata da seriati controlli emogasanalitici (o di saturazione/CO_2 transcutanea), nelle 4 ore antecedenti la dimissione.
- Minimo fabbisogno di ossigeno (FiO_2 < 0,35-0,45)
- Non necessita` di supporto inotropo, farmaci vasoattivi (vasodilatatori, vasocostrittori) o antiaritmici in infusione endovenosa continua.
- Aritmie controllate da almeno 24 ore.
- Rimozione dei monitoraggi emodinamici invasivi.
- Convulsioni controllate da almeno 24 ore.
- Pazienti che, in ventilazione non invasiva cronica, a seguito di una riacutizzazione respiratoria, siano ritornati alla condizione di base.
- Pazienti che, in trattamento dialitico peritoneale o in emodialisi, possano essere gestiti ambulatorialmente.

Questo documento è di proprietà dell'IRCCS "GIANNINA GASLINI", ogni riproduzione non autorizzata dallo stesso è vietata

- Pazienti con patologia a prognosi infausta quando, a seguito di una valutazione collegiale con coinvolgimento della famiglia e dello psicologo di reparto, si ritenga futile/accanimento terapeutico la prosecuzione delle cure in ambiente semi-intensivo.

5.7.2 Modalità operative del trasferimento

In caso di trasferimento dalla Terapia Semintensiva la procedura prevede:
a) Il reparto di destinazione viene identificato in base alla patologia di base ed alle competenze specialistiche necessarie alla prosecuzione delle cure.

b) Vengono presi contatti tra il medico della Terapia Semintensiva ed il medico della U.O. di destinazione e, ove pertinente, si procede ad una valutazione congiunta del paziente stesso. In caso di temporanea indisponibilità di posto letto presso il reparto di destinazione, sarà cura del medico del reparto stesso attivarsi per adeguare la recettività del reparto nel più breve tempo possibile. Preliminarmente al trasferimento il medico della Terapia Semintensiva predispone una sintetica epicrisi del caso, dove sia data evidenza dei problemi aperti e del programma di cura (terapie in atto). Il trasporto durante il trasferimento avviene a cura delle infermiere del reparto ricevente.
Deve essere predisposto un sistema di attento monitoraggio dei tempi di dimissione dal settore Semi-Intensivo, favorendo i flussi in uscita, nell'ottica di ottimizzare l'utilizzo delle risorse intensive disponibili in Istituto. Eventuali ritardi andranno segnalati tempestivamente alla Direzione Sanitaria e al Bed Manager

6. DISTRIBUZIONE E ARCHIVIAZIONE

La procedura è pubblicata nel Sistema Qualità Aziendale a cura del Servizio Qualità e ne viene data comunicazione mediante mail inviata a tutto Istituto.

7. RIFERIMENTI

- Standard JCI;
- Jaimovich DG: Admission and discharge guidelines for the pediatric patient requiring intermediate care. *Crit Care Med* 2004; 32:1215–1218;
- Guidelines for developing admission and discharge policies for the pediatric intensive care unit. *Crit Care Med* 1999; 27:843–845.

8. ALLEGATI

La procedura non è corredata di allegati.

Questo documento è di proprietà dell'IRCCS "GIANNINA GASLINI", ogni riproduzione non autorizzata dallo stesso è vietata
